# Effect of Host Size on Susceptibility of *Melanotus Communis* (Coleoptera: Elateridae) Wireworms to Entomopathogens

**DOI:** 10.2478/jofnem-2022-0033

**Published:** 2022-09-30

**Authors:** Livy Williams, Ron Cherry, David Shapiro-Ilan

**Affiliations:** 1 United States Department of Agriculture. Agricultural Research Service (USDA-ARS) U.S. Vegetable Laboratory, Charleston, SC 29414 US; 2 University of Florida, Everglades Research and Education Center, Wedgworth Laboratory, Belle Glade, FL 33430-4702 US; 3 USDA-ARS Southeastern Fruit and Tree Nut Research Station, Byron, GA 31008 US

**Keywords:** *Beauveria bassiana*, biological control, *Cordyceps javanica*, *Heterorhabditis bacteriophora*, host size, *Melanotus communis*, soil-borne, *Steinernema* spp., wireworms

## Abstract

Wireworms, the soil-borne larvae of click beetles (Coleoptera: Elateridae), are important crop pests throughout the world. In the eastern U.S., *Melanotus communis* larvae attack grain, root/ tuber, and vegetable crops. Our objectives were to characterize the pathogenicity and virulence of fungal and nematode entomopathogens on *M. communis* wireworms, and determine if wireworm size affected virulence. Pathogens tested included five entomopathogenic nematodes, *Steinernema carpocapsae* (All strain), *S. feltiae* (SN strain), *S. riobrave* (355 strain), *Heterorhabditis bacteriophora* (VS strain), and *H. indica* (HiHom1 strain); and two entomopathogenic fungi, *Beauveria bassiana* (GHA strain) and *Cordyceps javanica* (WF-GA17 strain). None of the pathogens tested caused >15% mortality at 7 or 14 days post-inoculation. Mortality was highest in *S. carpocapsae* (All strain); the other entomopathogens did not cause higher mortality than the untreated control. Overall, smaller wireworms were more susceptible than larger wireworms. Our results suggested that *M. communis* wireworms have defenses that limit the ability of the entomopathogens we tested to infect the wireworms. Conceivably, other entomopathogen strains or species may be more effective. Natural populations of entomopathogens may contribute to wireworm population reduction, but further studies are warranted before entomopathogens can be used for *M. communis* management.

*Melanotus communis* (Gyllenhal) (Coleoptera: Elateridae) is an important pest of maize, small grains, sugarcane, vegetables, and root and tuber crops in much of the United States east of the Rocky Mountains (Jansson and Seal, 1994; Lefko *et al*., 1998; Gill *et al*., 2014). Feeding on maize and small grains usually results in destruction of the plant and thus serious stand loss. In sugarcane, one wireworm per 1.5 m of sugarcane row can reduce stand and yield by 7.0% and 3.8%, respectively (Hall, 1990). In potato, *M. communis* damage has resulted in 45% downgrade of harvest (Jansson and LeCrone, 1991). Moreover, the feeding holes caused by wireworms in potato and sweetpotato facilitate attack by other invertebrates and pathogens. *M. communis* has a multiyear life cycle, during which larvae live in the soil for 2 yr to 6 yr (mediated partly by environmental conditions, such as soil factors, as well as type and availability of food) before emergence as adult click beetles in the spring and summer (Fenton, 1926). Mating and oviposition occur at this time, and eggs are laid singly in the soil or in crevices (Fenton, 1926; Cherry and Hall, 1986). Adult beetles cause little, if any, crop damage, but their dispersal and choice of oviposition sites contribute to location and severity of wireworm infestations (Hall, 1990; Cherry and Stansly, 2008; Gill *et al*., 2014; Cherry and Sandhu, 2020). Current control recommendations for *M. communis* include chemical insecticides, host plant resistance, and crop rotation practices, but these approaches are of limited effectiveness (Villani and Gould, 1985; Kuhar *et al*., 2003; Cherry and Nuessly, 2010; Traugott *et al*., 2015; Larson *et al*., 2016), thus research on development of alternative and/or complementary control strategies is warranted. Entomopathogens that live in the soil offer an attractive solution for the management of *M. communis* larvae.

In nature, entomopathogenic nematodes (EPNs) in the genera *Steinernema* Travassos and *Heterorhabditis* Poinar are obligate parasites of insects (Poinar, 1990; Grewal and Georgis, 1998). The EPN life cycle consists of an egg, four infective juvenile stages (IJs), and adults. EPNs ambush their hosts or engage in active host searching behavior (ShapiroIlan *et al*., 2018). These nematodes pass through one to three generations within the host, after which IJs, the free-living stage, exit the host cadaver and search for new hosts. The nematodes harbor mutualistic bacterial symbionts that play an important role in killing the host. EPNs are used as biological control agents against various pests, particularly soil-dwelling insects (Shapiro-Ilan *et al*., 2002, 2020; Lacey and Georgis, 2012). Recently developed biological control strategies for EPNs include “attract-and-kill” (la Forgia *et al*., 2021), combination of EPNs with chemicals (Mbata and Shapiro-Ilan, 2013), use of EPNs adapted to the same local conditions as the wireworm host (Morton and Garcia-del-Pino, 2007, 2009; Öğretmen *et al*., 2020), and genetic improvement of EPN strains through hybridization (Shapiro-Ilan *et al*., 1997). Under field conditions, EPNs can be applied with most standard agricultural equipment, thus facilitating their usefulness in pest management (Shapiro-Ilan and Dolinski, 2015).

Entomopathogenic fungi (EPF) have also shown promise for insect pest management (Lacey *et al*., 2015). EPF infection relies on physical contact with the host, after which conidia attach to the insect cuticle (aided by EPF-produced enzymes), grow into the host’s hemocoel, reproduce, and kill the host (Vega *et al*., 2012). Soil-borne EPFs are promising tools against wireworms (Van Herk and Vernon, 2011; Ritter and Richter, 2013). *Beauveria bassiana* (Bals.-Criv.) Vuill. and *Cordyceps javanica* are EPFs that live in the soil and have been effective in controlling numerous arthropods (Lacey *et al*., 2015). Milosavljevic *et al*. (2020) reported that the composition of entomopathogen communities had stronger effects on *Limonius californicus* wireworms than did species richness of the pathogens. In particular, a single entomopathogen species, *Metarhizium brunneum*, had stronger effects (greater host mortality and plant productivity) than did the other species tested (*B. bassiana, H. bacteriophora*, and *S. carpocapsae*). Recently developed strategies for use of EPFs include “attract-and-kill” (Brandl *et al*., 2017), integration of pathogen application on cover crops in the season prior to crop production (Reinbacher *et al*., 2021), and incorporation of EPFs as endophytes in plants (Vega, 2018).

The objectives of this study were to screen EPNs and EPFs on *M. communis* larvae and to determine the effect of host size (based on larval weight) on susceptibility to these pathogens. To our knowledge, EPF and EPN biocontrol agents have not been tested for pathogenicity or virulence to *M. communis*.

## Materials and Methods

### Insect and pathogen collection and storage

Wireworms (larvae) were collected from November 2020 to April 2021 by digging under sugarcane stools (plants) at the Everglades Research and Education Center, University of Florida, in Palm Beach County, Florida. These collections included wireworms of all sizes because multiple generations of *M. communis* occur in the soil; thus, it is expected to find a range of size classes. After collection, wireworms were identified (Riley and Keaster, 1979) and stored at 18°C in moist soil with carrot pieces for food before shipment to Georgia. Prior to shipment, wireworms were placed in containers with moist paper towels to provide a substrate and moisture and carrots for food. These containers were then shipped overnight (USDA-APHIS permit no. P526P-19-02621 to Shapiro-Ilan) to the USDA-ARS Southeast Fruit and Tree Nut Laboratory, Byron, Georgia. After arrival, the wireworms were held in darkness at 18°C. Before the assays, the wireworms were checked for morbidity/ mortality/onset of molting (based on coloration and reaction to prodding), and healthy nonmolting individuals were recounted. At this time, wireworms were sorted with a digital balance into two size classes based on weight (small = 0.01 g to 0.06 g; large = 0.07 g to 0.12 g).

All pathogens were obtained from the USDA-ARS collection in Byron, Georgia. The following pathogens were tested for virulence to *M. communis*: two EPFs, viz., *B. bassiana* (Bals.-Criv.) Vuill. (Hypocreales: Cordycipitaceae) (GHA strain) and *C. javanica* (Frieder. & Bally) Kepler, B. Shrestha & Spatafora (Hypocreales: Cordycipitaceae) (WF-GA17 strain); and five EPNs, viz., *S. carpocapsae* (Weiser) (strain All), *S. feltiae* (Filipjev) (SN strain), *S. riobrave* Cabanillas, Poinar, and Raulston (strain 355), *H. bacteriophora* Poinar (VS strain), and *H. indica* Poinar, Karunakar, and David (Hom1 strain). We chose these EPNs because they are all commercially available and hence could conceivably be used in biocontrol applications if the results supported such action.

Each fungal isolate was cultured on parafilm-wrapped potato dextrose agar (PDA) petri plates (100 mm diameter) and incubated at 25°C at light:dark cycle of 14:10 hr. Fungi were scraped from the inoculated plates 7 d to 10 d postincubation, and the resulting conidia were placed inside conical tubes (50 ml) that contained 30 ml of sterile 0.05% Silwet L-77 (Momentive Performance Materials Inc., Waterford, NY) solution. Eight glass beads were added to each tube and vortexed for approximately 5 min, and the desired concentration (1 × 10^8^ conidia ml^-1^) was determined using a hemocytometer under the microscope. Conidial viability of each isolate was assessed by plating 0.1 ml of solution of each of the two isolates on small Sabouraud dextrose agar yeast (SDAY) petri plates (60 mm diameter) (Inglis *et al*., 2012) and incubated at 25°C in the dark. Percent germination was determined under the microscope at 16 hr postincubation by assessment of 200 spores from each plate. A total of four counts (two counts from each plate) were taken per fungal isolate, after which the required concentration was adjusted according to the germination rate (%) of each isolate (Usman *et al*., 2020).

All nematodes were reared in parallel on the last instar larvae of *Galleria mellonella* (L.) (Sunfish Bait, Webster, WI) at 25°C, and the IJs were collected on White traps (White, 1927; Shapiro-Ilan *et al*., 2016). Nematodes were stored in tissue culture flasks (250 ml) at 13°C for no longer than 3 wk before experimentation.

## Experimentation

Effect of entomopathogens on *M. communis* was assessed in two separate trials using a randomized complete block design and consisted of seven treatments (two EPFs and five EPNs) plus a control group. Experimental arenas consisted of plastic cups (30 ml) that were filled with 20 g of soil giving a surface area of approximately 28 cm^2^. The soil was obtained from the USDA-ARS pecan orchard (Byron, Georgia); it was loamy sand (84% sand, 10% silt, and 6% clay) with 2.8% organic matter and a pH of 6.1. Prior to experimentation, the soil was autoclaved (275°C for 30 min) and held at room temperature for at least 2 wk.

Larval size of *M. communis* was tested in both trials (small vs large). In each trial, three replicates of each treatment were tested, and each replicate consisted of five larvae in individual cups. These larvae ware added to cups with moistened soil and a 2.5-cm piece of carrot. Wireworms that did not burrow into the soil within 12 hr were replaced. Treatments were subsequently added in 1 ml suspensions; the final soil moisture level was at field capacity (16%). The treatments were pipetted onto the soil surface, and the control cups received only 1 ml of tap water. The concentration of EPFs was 10^8^ viable conidia per milliliter and, for EPNs, the concentration was 2,800 IJs (100 IJs/cm^2^). These rates were chosen because they are comparable to field rates used for the pathogens tested (Lacey *et al*., 2015; Shapiro-Ilan *et al*., 2020). The cups were gently flipped three times to aid dispersion, placed on a tray with moist towels, and held in an incubator (25°C) in darkness. Wireworm mortality was assessed twice, i.e., at 7 d and 14 d postapplication. During the interval between these times, wireworms were held in darkness at 25°C.

### Data analysis

Based on residual plots, mortality data recorded as percentages were transformed (arcsine of the square root) before analysis. Using a factorial design with treatment and wireworm size as the main factors, data were analyzed using PROC GLM (SAS Institute Inc., 2016). Within each trial, when no significant interaction was detected between trial and treatment or between trial and wireworm size (*P* > 0.05), data were pooled among trials. Multiple treatment differences were elucidated with Tukey’s studentized range (honestly significant difference [HSD]) test (SAS Institute Inc., 2016).

## Results and Discussion

**Figure 1:**
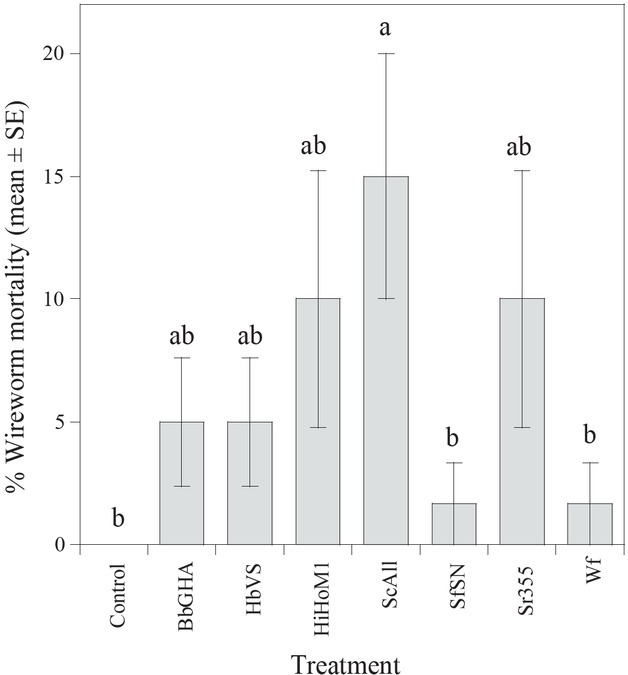
Mean (% + SE) mortality of *Melanotus communis* larvae when treated with *Beauveria bassiana* (GHA strain), *Cordyceps javanica* (WF-GA17 strain), *Steinernema carpocapsae* (strain All), *S. feltiae* (SN strain), *S. riobrave* (strain 355), *Heterorhabditis bacteriophora* (VS strain), and *H. indica* (Hom1 strain) in a laboratory small cup bioassay. Treatment means with the same letter are not significantly different (*P* > 0.05, Tukey’s HSD test). HSD, honestly significant difference; SE, standard error of the mean.

**Figure 2:**
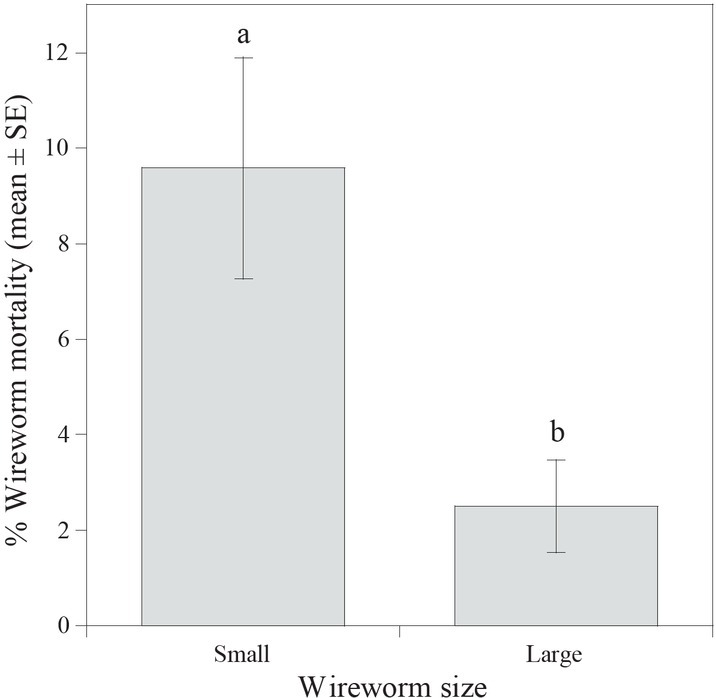
Mean (% + SE) mortality of small and large *Melanotus communis* larvae when treated with *Beauveria bassiana* (GHA strain), *Cordyceps javanica* (WF-GA17 strain), *Steinernema carpocapsae* (strain All), *S. feltiae* (SN strain), *S. riobrave* (strain 355), *Heterorhabditis bacteriophora* (VS strain), and *H. indica* (Hom1 strain) in a laboratory small cup bioassay. Different letters above the bars indicate statistical significance (*P* < 0.05). SE, standard error of the mean.

**Figure 3:**
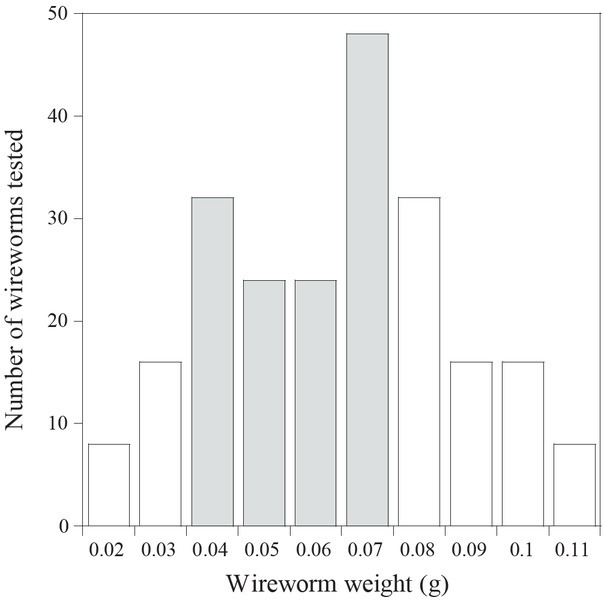
Frequency distribution of wireworm weights tested in both trials. Shaded columns denote range of wireworm weights in which mortality was observed (0.04 g to 0.07 g).

Given that there was no interaction between main factors (*F =* 1.05; df = 7, 72; *P* = 0.407), wireworm size and treatment effects were analyzed independently. In trials with wireworms of both size classes, significant differences (*F* = 2.95; df = 7, 72; *P* = 0.0089) were detected among the treatments. Treatment mortality ranged from 1.7% to 15% and was the highest in *S. carpocapsae* (strain All) (Fig. 1). In trials comparing size classes of wireworms across all treatments, greater mortality was observed in small wireworms (0.04 g to 0.07 g) than in large wireworms (*F* = 10.1; df = 1, 72; *P* = 0.0021) (Fig. 2). Figure 3 presents the frequency distribution of the mass of wireworms used in both trials and shows the range of sizes (0.04 g to 0.07 g) in which mortality was observed.

Our results revealed that *S. carpocapsae* (strain All) was the only treatment found to be pathogenic to *M. communis* larvae in small cup laboratory bioassays; mortality observed in the other pathogen treatments was not significantly different from that in the control. While we measured only mortality for assessment of pathogenicity, the pathogens in our treatments may have indirectly affected wireworm feeding, growth, or other aspects of fitness. Overall mortality levels in *M. communis* larvae were low compared to similar studies with the same pathogens on other insect taxa, with reported mortality ranging from 25% to 80% (Shapiro-Ilan *et al*., 1999; Shapiro-Ilan, 2001; Shapiro-Ilan *et al*., 2002; Gulzar *et al*., 2021). Ansari *et al*. (2009) reported wide variation in virulence between different species and strains of EPFs and EPNs to *A. lineatus* wireworms. This host was most susceptible to *Metarhizium anisopliae* (mortality >90%), was moderately susceptible (mortality 67%) to the EPN *H. bacteriophora*, and was not significantly affected by *B. bassiana*. *M. brunneum* has shown promise in the management of *Agriotes* spp. using an “attract-and-kill” strategy (Brandl *et al*., 2017) and on application to cover crops (Reinbacher *et al*., 2021). The variability and low overall virulence observed in some pathogen/wireworm studies (Morton and Garcia-del-Pino, 2017; Sandhi *et al*., 2020; Sandhi *et al*., 2021), including the current study, may be due to the inability of the pathogen to enter the wireworm’s body through either natural openings or intersegmental membrane, in the case of nematodes (Eidt and Thurston, 1995), or via the cuticle, in the case of fungi (Zhang *et al*., 2014). Immune responses of wireworms also serve as a defense against EPN infection (Eidt and Thurston, 1995; Rahatkhah *et al*., 2015). Additionally, using entomopathogen strains adapted to the same local conditions as the host may lead to improved virulence (Campos-Herrera and Gutiérrez, 2009; Lacey and Georgis, 2012).

Little is known about the effects of wireworm size on pathogenicity to EPNs and EPFs. The current study found that *M. communis* wireworms that we classified as small (based on larval weight) are more susceptible to entomopathogens than the larger wireworms. Contrary to our study, Van Herk and Vernon (2011) reported that larger *A. obscurus* wireworms were more susceptible to *M. anisopliae* than smaller wireworms. Studies with other insect taxa indicate that susceptibility of insects to pathogens can be affected by host size (Smits *et al*., 1994; Ansari *et al*., 2009; Power *et al*., 2009). First instar *Popillia japonica* larvae were more susceptible to *H. bacteriophora* than were third instar larvae (Powers *et al*., 2009). Conversely, Smits *et al*. (1994) reported that susceptibility of *Phyllopertha horticola* L. (Coleoptera: Scarabaeidae) to *Heterorhabditis* spp. and *Steinernema* spp. increased with larval development. Interestingly, in our study, the very-small-sized *M. communis* (0.02 g and 0.03 g) were also not susceptible to infection. Bastidas *et al*. (2014) reported that very small hosts (“microhosts”) are difficult for EPNs to infect, and thus it appears there is an optimum threshold size. It is likely that wireworm size interacts with other factors, such as soil type, soil moisture, wireworm physiological state, and pathogen species/strain to determine susceptibility to entomopathogens.

Our findings indicate that further work is necessary before a management strategy relying on entomopathogens can be developed for *M. communis*. Research areas with potential include exploration for entomopathogens more closely linked to *M. communis* larvae (Campos-Herrera and Gutiérrez, 2009; Lacey and Georgis, 2012). The fact that some strains or species of soil-borne entomopathogens are quite efficacious on wireworms, while others have negligible effects, suggests a close evolutionary relationship between wireworms and their pathogens. A deeper understanding of this relationship may lead to improved *M. communis* wireworm management practices. Genetic improvement of pathogen strains through hybridization is promising (Shapiro *et al*., 1997; Shapiro-Ilan *et al*., 2020) and may yield improved virulence against wireworms. Moreover, basic studies are needed to better understand wireworm defense mechanisms against pathogens, evaluation of pathogens used in combination (i.e., additive or synergistic effects) (Shapiro-Ilan *et al*., 2004), and use of natural boosters or synthetic additives to enhance the efficacy of pathogens by reducing the effectiveness of wireworm defenses (Oliveira-Hofman *et al*., 2019). Use of entomopathogens in combination with synthetic chemicals also has potential for wireworm control (Mbata and Shapiro-Ilan, 2013). Additionally, the potential for entomopathogens to contribute to population regulation in conservation biocontrol approaches can be explored (Jaffuel *et al*., 2017; Reinbacher *et al*., 2021).
